# Live-Cell FRET Imaging Reveals a Role of Extracellular Signal-Regulated Kinase Activity Dynamics in Thymocyte Motility

**DOI:** 10.1016/j.isci.2018.11.025

**Published:** 2018-11-20

**Authors:** Yoshinobu Konishi, Kenta Terai, Yasuhide Furuta, Hiroshi Kiyonari, Takaya Abe, Yoshihiro Ueda, Tatsuo Kinashi, Yoko Hamazaki, Akifumi Takaori-Kondo, Michiyuki Matsuda

**Affiliations:** 1 Department of Pathology and Biology of Diseases, Graduate School of Medicine, Kyoto University, Kyoto 606-8501, Japan; 2 Laboratory of Bioimaging and Cell Signaling, Graduate School of Biostudies, Kyoto University, Kyoto 606-8501, Japan; 3 Laboratories for Animal Resource Development and Genetic Engineering, RIKEN Center for Biosystem Dynamics Research, Kobe 650-0047, Japan; 4 Department of Molecular Genetics, Institute of Biomedical Science, Kansai Medical University, Osaka 570-8506, Japan; 5 Department of Life Science Frontiers, Center for iPS Cell Research and Application, Kyoto University, Kyoto 606-8507, Japan; 6 Laboratory of Immunobiology, Graduate School of Medicine, Kyoto University, Kyoto 606-8507, Japan; 7 Department of Hematology and Oncology, Graduate School of Medicine, Kyoto University, Kyoto 606-8507, Japan

**Keywords:** Biological Sciences, Components of the Immune System, Molecular Biology

## Abstract

Extracellular signal-regulated kinase (ERK) plays critical roles in T cell development in the thymus. Nevertheless, the dynamics of ERK activity and the role of ERK in regulating thymocyte motility remain largely unknown due to technical limitations. To visualize ERK activity in thymocytes, we here developed knockin reporter mice expressing a Förster/fluorescence resonance energy transfer (FRET)-based biosensor for ERK from the *ROSA26* locus. Live imaging of thymocytes isolated from the reporter mice revealed that ERK regulates thymocyte motility in a subtype-specific manner. Negative correlation between ERK activity and motility was observed in CD4/CD8 double-positive thymocytes and CD8 single-positive thymocytes, but not in CD4 single-positive thymocytes. Interestingly, however, the temporal deviations of ERK activity from the average correlate with the motility of CD4 single-positive thymocytes. Thus, live-cell FRET imaging will open a window to understanding the dynamic nature and the diverse functions of ERK signaling in T cell biology.

## Highlights

• Mice expressing EKAREV from *ROSA26* locus enable ERK activity monitoring in T cells
• ERK activity negatively regulates the motility of thymocytes in the thymus
• Temporal dynamics of ERK activity regulates cell motility of CD4-SP in the medulla
• TCR signal from intercellular association induces ERK activity dynamics in CD4-SP

## Graphical Abstract

**Figure:**
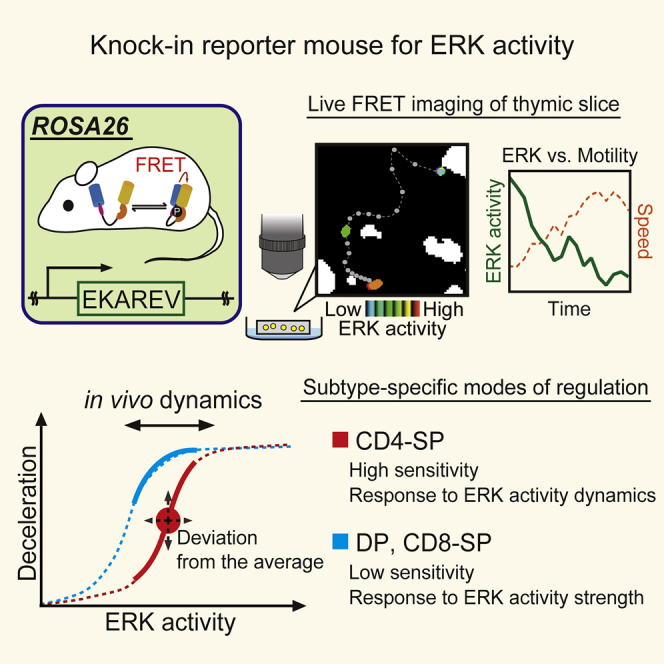

## Introduction

In the immune system the thymus plays a central role as the primary lymphoid organ for the development and differentiation of T cells (Lancaster et al., 2017, Miller, 2014, Nadia and Robey, 2016). Thymocyte maturation is a dynamic process that takes place in two distinct spatial compartments of the thymus, the cortex and the medulla. Thymocytes migrate through these compartments and engage in cellular interactions essential for their differentiation into functional and self-tolerant T cells (Penit and Vasseur, 1988). The thymic cortex harbors immature CD4/CD8 double-positive (DP) thymocytes expressing a stochastically rearranged T cell receptor (TCR). A fraction of DP thymocytes successfully receive TCR-activating signals for their positive selection through interaction with major histocompatibility complex (MHC) molecules on cortical thymic epithelial cells (TECs) (Savage and Davis, 2001). The positively selected DP thymocytes then undergo commitment to the CD4 or CD8 single-positive (SP) T cell lineage and migrate to the thymic medulla (Bosselut et al., 2003, Keefe et al., 1999), where autoreactive thymocytes are eliminated by negative selection (Cosway et al., 2017, Kappler et al., 1987). The commitment to the CD4-SP and CD8-SP subsets depends on TCR interaction with MHC classes II and I, respectively (Teh et al., 1988).

Several lines of evidence suggest that extracellular signal-regulated kinase (ERK) serves as a principal signal transducer downstream of TCR in multiple T cell developmental or differential processes (Kortum et al., 2013). Indeed, mice deficient for two isoforms of ERK, ERK1 and ERK2, exhibit defective pre-TCR-driven proliferation and TCR-mediated positive selection (Fischer et al., 2005). Moreover, differential spatiotemporal activation of ERK signaling has been implicated in positive and negative selection in the thymus (Daniels et al., 2006, McNeil et al., 2005). The duration of ERK activation has also been shown to play an important role in CD4 versus CD8 lineage commitment (Sharp et al., 1997, Wilkinson and Kaye, 2001).

Two-photon time-lapse imaging of living thymic tissue has revealed high motility of developing T cells within the three-dimensional (3D) environment of the thymus (Bousso et al., 2002, Witt et al., 2005). Thus, elucidation of the mechanisms by which developing thymocytes traffic through and interact with the thymic microenvironment will enhance our understanding of T cell differentiation (Campbell et al., 1999, Ross et al., 2014, Ki et al., 2017; Ueda et al., 2016). Despite its obvious importance, the dynamics of ERK activity and its role in regulating cell motility within the thymic microenvironment remain largely unknown due to technical limitations. Traditional approaches assessing the phosphorylation states of ERK, such as immunoblotting, flow cytometric analysis, and immunohistochemistry, cannot reveal the role of ERK in the cell motility within their native environment. Therefore, there is an increasing demand for methods to observe ERK activity under physiological conditions in real time.

To meet this demand, we and others are using biosensors based on the principle of Förster/fluorescence resonance energy transfer (FRET) (Aoki et al., 2013b, Hoekstra et al., 2015, Miyawaki and Niino, 2015). FRET is the process by which a donor fluorophore in an excited state transfers energy to a neighboring acceptor fluorophore, thereby causing the acceptor to emit fluorescence at its characteristic wavelength. EKAREV is a highly sensitive genetically encoded intramolecular FRET biosensor for ERK activity, which has been contributing to our understanding of the spatiotemporal dynamics of ERK signaling in living cells (Harvey et al., 2008, Komatsu et al., 2011). Phosphorylation of the substrate peptide by ERK promotes a conformational change of EKAREV to increase the FRET efficiency from a donor cyan fluorescent protein (CFP) to an acceptor yellow fluorescent protein (YFP). The fluorescence ratio of YFP to CFP, hereinafter the FRET/CFP ratio, is used to represent FRET efficiency (ERK activity). The recent development of transgenic mice expressing EKAREV, called Eisuke mice, has further paved the way to the observation of molecular activities in live animals (Kamioka et al., 2012). Indeed, this development has realized the intravital imaging of ERK activities in organs including the brain, skin, and intestines (Hiratsuka et al., 2015, Muta et al., 2018, Ogura et al., 2018, Yamaguchi et al., 2015). However, such imaging has yet to be achieved for T cells because of the low expression level of EKAREV in lymphocytes of the Eisuke mice.

The goal of this study is to unravel the potential cross talk between ERK activity dynamics and cell motility within the thymic microenvironment. To this end, we have developed knockin reporter mouse lines expressing EKAREV from the *ROSA26* locus. Live imaging of thymocytes *in situ* has revealed that ERK activation suppresses thymocyte motility within the thymic microenvironment. Interestingly, we have revealed two different modes of translating ERK activity dynamics into cell motility in a manner dependent on cell types. The strength of ERK activity correlates negatively with cell motility in both the DP and CD8-SP subsets, whereas temporal deviations of ERK activity correlate with cell motility in the CD4-SP subset. These results suggest that cell motility of CD4-SP is more sensitive to ERK activity dynamics compared with the motility of other subsets under physiological conditions. Thus, the live-cell FRET imaging of ERK activity will open a window to understanding the dynamic nature and the diverse functions of ERK signaling in T cell biology.

## Results

### Lck-EKAREV-NLS Mice Enable ERK Activity Monitoring in T Cells

**Figure 1:**
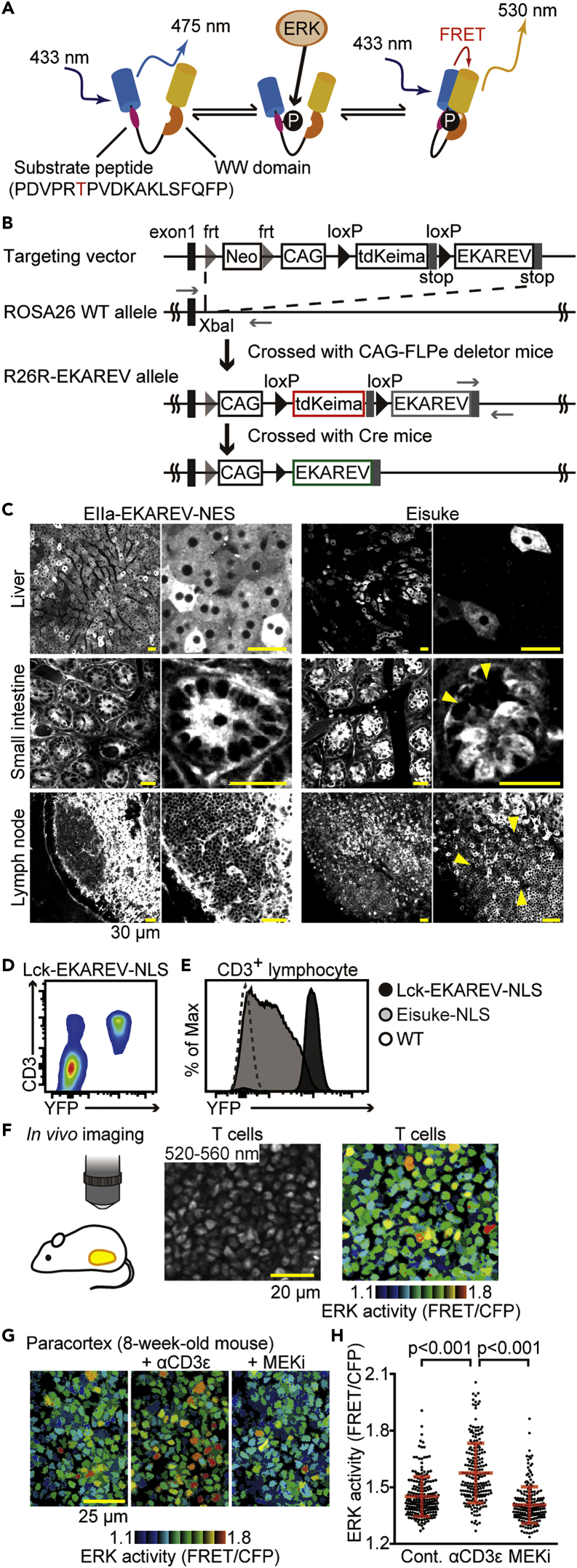
Lck-EKAREV-NLS Mice Enable ERK Activity Monitoring in Lymphocytes (A) A schema of EKAREV. Phosphorylation of the substrate peptide induces a conformational change and a concomitant increase in the FRET efficiency. (B) A schema of the generation of R26R-EKAREV mice. Top to bottom: the structure of the targeting vector, the wild-type *ROSA26* locus with the location of the insertion site, the structure of the *R26R*-EKAREV allele after flippase-frt-mediated excision of the *frt*-flanked neo cassette, and the structure of the *R26*-EKAREV allele after Cre-loxP-mediated excision of the *loxP*-flanked *tdKeima* sequence. Fragments shown in red and green can be expressed. The black rectangles on the left indicate the location of the first exon of the non-coding RNA in the *ROSA26* locus. The gray rectangles indicate the location of the stop codons. *loxP* sequences are indicated by black arrowheads. *frt* sequences are indicated by gray arrowheads. Neo is the neo cassette. DT-A is a diphtheria toxin A fragment gene for negative selection. (C) Representative fluorescence images of EIIa-EKAREV-NES (left) and Eisuke (right) through a BA 520-560 nm filter shown in grayscale. The excitation wavelength was 840 nm. Top to bottom: the liver, the small intestine, and the lymph node. Left to right: image of EKAREV fluorescence and enlarged view of the left image. The yellow arrowheads indicate the regions with the *CAG* promoter being inactive or only weakly active. Scale bar, 30 μm. (D) Flow cytometric profile of EKAREV and CD3 expression among lymphocytes obtained from the lymph node of Lck-EKAREV-NLS. EKAREV expression is represented by YFP intensity. (E) Flow cytometry of EKAREV-NLS expression in CD3^+^ lymphocytes of the lymph nodes derived from C57BL/6 (WT), Eisuke-NLS, and Lck-EKAREV-NLS mice. (F) Images of the paracortex region of the lymph node in a living mouse obtained by TPEM as shown in the schema. (Left) Fluorescence image of T cells through a BA 520-560 nm emission filter. (Right) FRET/CFP ratio image shown in the intensity-modulated display (IMD) mode. Scale bar, 20 μm. (G) Representative FRET/CFP ratio images of the T cells in the paracortex shown in IMD mode. Time-lapse imaging of T cells in the paracortex is performed for 90 min. Anti-CD3ɛ antibody (50 μg/body) was injected intravenously at 0 min. After 60 min, MEK inhibitor (PD0325901) (100 μg/body) was injected intravenously. The age of mouse in weeks is indicated. Left to right: FRET/CFP ratio image obtained just before anti-CD3ɛ antibody administration, 60 min after anti-CD3ɛ antibody administration, and 30 min after MEK inhibitor administration. Scale bar, 25 μm. (H) The FRET/CFP ratio just before anti-CD3ɛ antibody administration (n = 211 cells), 60 min after anti-CD3ɛ antibody administration (n = 212 cells), and 30 min after MEK inhibitor administration (n = 207 cells). Similar experiments were performed with two independent mice and shown in Figure S1. Dots indicate the FRET/CFP ratio in each T cell. All data are presented as mean ± SD. p values were calculated by Student's two-sample t test.

EKAREV is a genetically encoded intramolecular FRET biosensor for monitoring ERK activity in living cells (Figure 1A) (Komatsu et al., 2011). EKAREV-NLS and EKAREV-NES contain a nuclear localization signal and a nuclear export signal, respectively. In the first generation of transgenic mice, EKAREV was barely expressed in lymphocytes and gene silenced in some tissues. To express EKAREV ubiquitously, we introduced the cDNAs of EKAREV-NLS and EKAREV-NES into the *ROSA26* locus (Figure 1B) to generate knockin reporter mouse lines named Gt(ROSA)26Sor^tm1(CAG-loxP-tdKeima-loxP-EKAREV-NES)^ and Gt(ROSA)26Sor^tm1(CAG-loxP-tdKeima-loxP-EKAREV-NLS)^ (hereinafter called R26R-EKAREV-NES and R26R-EKAREV-NLS), respectively. These mouse lines are designed to express the tdKeima fluorescent protein before Cre-mediated excision and EKAREV after excision, under the CAG promoter in the *ROSA26* locus.

To obtain the proof of concept, we crossed R26R-EKAREV-NES with EIIa-Cre mice, in which Cre is expressed at the zygote stage (Lakso et al., 1996), and generated a mouse line named EIIa-Cre/R26R-EKAREV-NES (hereinafter called EIIa-EKAREV-NES). The tissue distribution of EKAREV in EIIa-EKAREV-NES was compared with that of the previously reported Eisuke mouse, the transgenic mouse expressing EKAREV, by intravital imaging of the liver, the small intestine, and the lymph node (Figure 1C). In the liver of Eisuke mice, EKAREV-NES was expressed in a mosaic pattern. A similar observation was made in the crypt of the intestine and the lymph nodes, suggesting gene silencing of the EKAREV-NES. In contrast, EKAREV-NES was expressed in all hepatocytes, intestinal cells, and lymphocytes in EIIa-EKAREV-NES mice as anticipated.

To study ERK activity dynamics in T cells, R26R-EKAREV-NLS mice were crossed with Lck-Cre mice to generate Lck-Cre/R26R-EKAREV-NLS (hereinafter called Lck-EKAREV-NLS). To validate EKAREV-NLS expression in T cells, lymphocytes obtained from inguinal lymph nodes of EKAREV-NLS were analyzed by flow cytometry (Figure 1D). EKAREV expression was limited to CD3^+^ cells, and more than 95% of CD3^+^ cells expressed EKAREV, indicating that EKAREV expression was T cell specific. The expression level of EKAREV in CD3^+^ cells was markedly increased in Lck-EKAREV-NLS mice compared with the Eisuke-NLS mice (Figure 1E). Similar results were obtained with CD3^+^ cells derived from the spleen (data not shown).

We next visualized ERK activity in the lymph node by two-photon excitation microscopy (TPEM). In the paracortex region, where T cells are packed at high density, the FRET/CFP ratio in T cells was considerably heterogeneous, suggesting that ERK activity was significantly different among the individual T cells (Figure 1F). Upon intravenous administration of anti-CD3ɛ antibody, which activates the TCR signaling pathway, the FRET/CFP ratio was increased rapidly and remarkably. Following injection of PD0325901, an MEK inhibitor, the FRET/CFP ratio was significantly reduced (Figures 1G and 1H; Video S1). Two similar results were obtained by using two additional mice (Figure S1). These results indicate that EKAREV-NLS faithfully represents ERK activity in T cells *in vivo*.

Video S1. *In Vivo* Imaging of T Cells after Antibody Injection, Related to Figure 1 *In vivo* imaging of T cells in the paracortex region of the lymph node. This movie shows the ERK activation in T cells after anti-CD3ɛ antibody (50 μg/body) injection at 0 min. Scale bars, 10 μm. Time is shown in minutes.

### FRET Imaging of Explanted Thymic Lobes Visualizes Heterogeneous ERK Activity of Cortical Thymocytes

**Figure 2:**
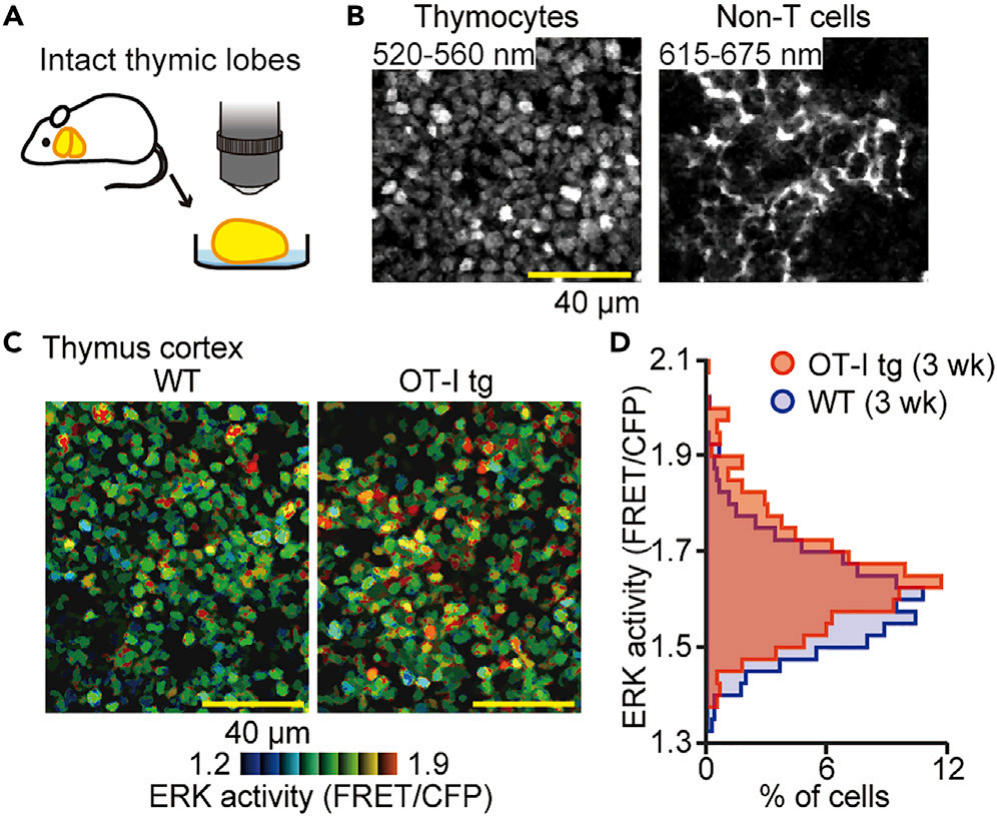
FRET Imaging of Explanted Thymic Lobes Visualizes the Heterogeneous ERK Activity of Cortical Thymocytes (A) The experimental design for analyzing the ERK activity of thymocytes in intact thymic lobes. The thymic lobes isolated from Lck-EKAREV-NLS were examined by TPEM. (B) Representative images of the thymic cortex. (Left) Fluorescence image of thymocytes through a BA 520-560 nm emission filter. (Right) Fluorescence image through a BA 615-675 nm emission filter showing non-T cells, including TECs. The excitation wavelength was 840 nm. Scale bar, 40 μm. (C) Representative FRET/CFP ratio image of WT thymocytes (left) and OT-I tg thymocytes (right) in the cortex shown in intensity-modulated display (IMD) mode. Scale bar, 40 μm. (D) The relative frequency of the FRET/CFP ratio of WT thymocytes (blue; n = 829 cells) and OT-I tg thymocytes (red; n = 721 cells). The age of mice in weeks are indicated. Similar experiments were performed with two independent mice and shown in Figure S2.

After the initial characterization of Lck-EKAREV-NLS mice, we attempted to visualize the ERK activity of thymocytes in the thymic microenvironment. Long-term *in vivo* microscopy of the thymus is difficult due to the anatomical location abutting the heart. To circumvent this problem, we adopted *ex vivo* thymic lobe imaging (Figure 2A). In the thymic cortex of Lck-EKAREV-NLS mice, thymocytes expressing EKAREV were detected through a BA 520-560 nm emission filter at the excitation wavelength of 840 nm, whereas the other cells expressing tdKeima, including TECs, were detected through a BA 615-675 nm emission filter (Figure 2B). Like the ERK activity of T cells in the lymph node, the ERK activity of thymocytes was heterogeneous (Figure 2C, left). This heterogeneity was remindful of a similar heterogeneous ERK activity observed in tissue culture cells and intestinal epithelial cells (Albeck et al., 2013, Aoki et al., 2013a, Muta et al., 2018). In these previous reports, the heterogeneous ERK activity was evoked by spontaneous ERK activation pulses of approximately 15–30 min duration. In the present study, however, we rarely observed similar spontaneous ERK activation pulses in thymocytes over the 20 min imaging (Video S2), indicating that the mechanism underlying the heterogeneous ERK activity is different between epithelial cells and thymocytes.

Video S2. Live Imaging of Thymocytes Migrating in the Cortex, Related to Figure 2 Live-cell FRET imaging of cortical thymocytes in the intact thymic lobe. This movie shows the dynamics of ERK activity for 20 min. Scale bars, 10 μm. Time is shown in minutes.

It has been shown that ERK activation is required for positive selection of T cells in the thymus (Daniels et al., 2006, Fischer et al., 2005, McNeil et al., 2005). Therefore, the heterogeneous ERK activity may suggest the different stages of positive selection or different strength of TCR-MHC interactions. To examine this possibility, we crossed Lck-EKAREV-NLS mice with ovalbumin (OVA)-specific TCR transgenic (OT-I) mice and then examined the ERK activity of thymocytes within thymic lobes by TPEM (Figure 2C, right). The thymus was explanted from mice without any pretreatment, including OVA challenge. Compared with Lck-EKAREV-NLS thymocytes (wild-type [WT] thymocytes), thymocytes with OT-I TCR (OT-I tg thymocytes) exhibited higher ERK activity with a similar degree of heterogeneity (Figures 2D and S2). The majority of OT-I tg thymocytes expressed a single TCR that could be positively selected by MHC class I molecules on the cortical TECs. Therefore, this result suggests that the TCR-MHC interaction induced the upward shift of ERK activity among cortical thymocytes and that the heterogeneous ERK activity was not just the result of the different strength of TCR-MHC interactions.

### Environmental Cues in the Thymus Cause High Basal ERK Activity in Thymocytes

**Figure 3:**
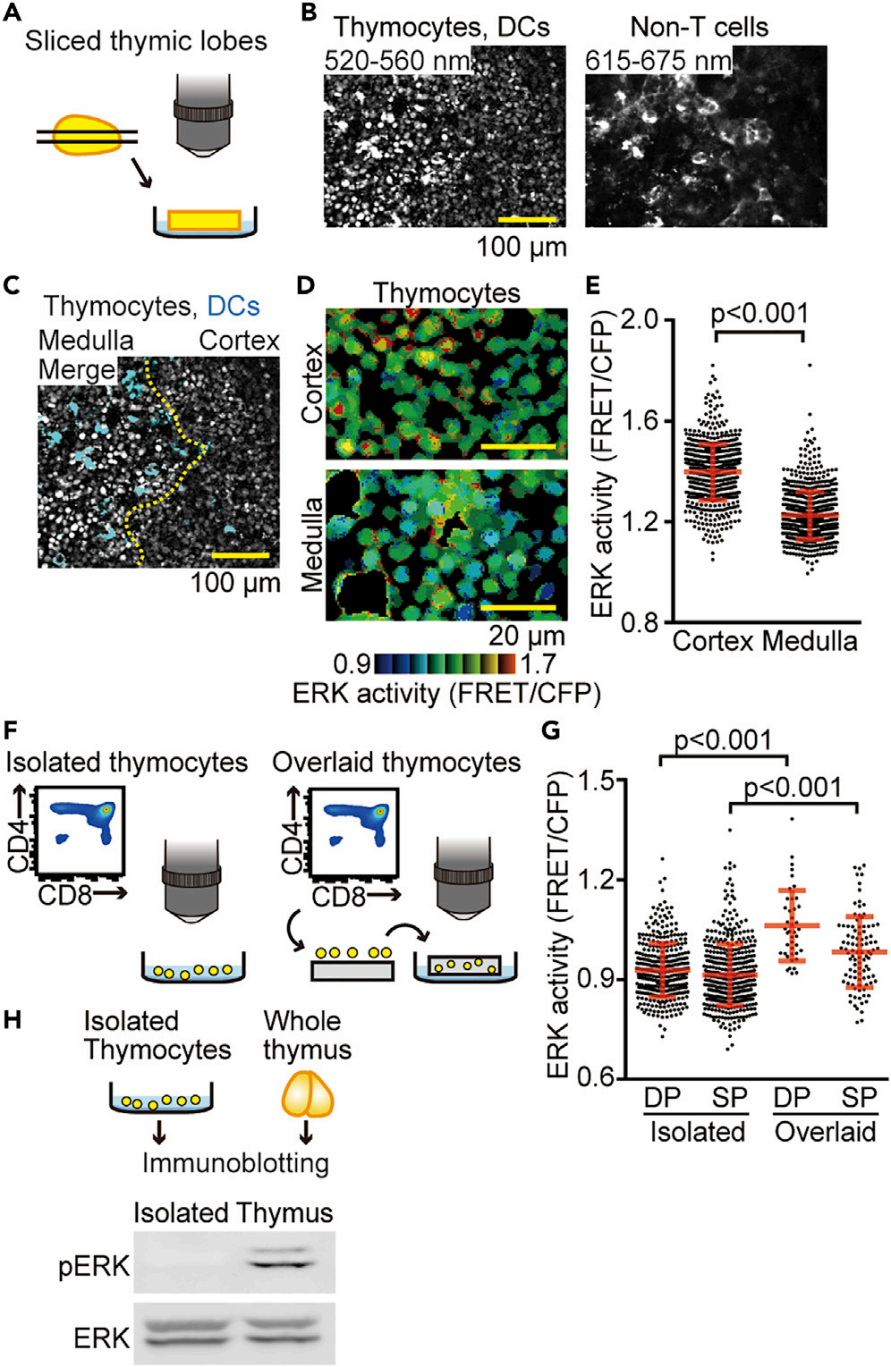
Environmental Cues in the Thymus are Required for the Basal ERK Activity of Thymocytes (A) The experimental design for analyzing ERK activity in the sliced thymic lobe. The thymus isolated from Lck-EKAREV-NLS mice was mounted in agarose and sliced with a vibratome. Then, the sliced thymic lobes were observed by TPEM. (B) Representative images of sliced thymic lobes. (Left) Fluorescence image through a BA 520-560 nm emission filter showing both thymocytes and thymic DCs. (Right) Fluorescence image through a BA 615-675 nm emission filter showing non-T cells, including thymic DCs. Scale bar, 100 μm. (C) A representative merged image (merged fluorescence images through a BA 520-560 nm emission filter and BA 615-675 nm emission filter) showing the cortical medullary junction. Images shown in (B) were merged. The relative density of inherent autofluorescence of thymic DCs (cyan) was used to identify the cortical-medullary junction (dashed yellow line). Scale bar, 100 μm. (D) Representative FRET/CFP ratio images of the cortex (top) and the medulla (bottom) in intensity-modulated display (IMD) mode. Scale bar, 20 μm. (E) The FRET/CFP ratio examined within sliced thymic lobes. Dots indicate the FRET/CFP ratio in each thymocyte. Data represent the analysis of samples from three individual mice (n = 676 cells for the cortex and n = 766 cells for the medulla). See also Table S1. All data are presented as mean ± SD. p value was calculated by Student's two-sample t test. (F) The experimental design for monitoring the ERK activity of specific subsets of isolated thymocytes *in vitro* (left) and thymocytes overlaid onto sliced thymic lobes (right). For the observation of isolated thymocytes *in vitro*, thymocytes from Lck-EKAREV-NLS mice were sorted and directly imaged by TPEM. For the observation of overlaid thymocytes, sorted subsets of thymocytes were overlaid onto sliced thymic lobes obtained from C57BL/6 mice. Thymocytes on the slices were incubated for at least 3 hr at 37°C/5% CO_2_ to allow cells to infiltrate the tissue. Then, the sliced thymic lobes were examined by TPEM. (G) FRET/CFP ratios of the indicated subsets of isolated thymocytes *in vitro* and of thymocytes overlaid on the sliced thymic lobes. Each dot indicates the FRET/CFP ratio of each thymocyte. Data represent the analysis of samples from three individual mice for isolated thymocytes *in vitro* (indicated as Isolated) (n = 382 cells for DP and n = 474 cells for SP). Data are from the analysis of samples from two individual mice for thymocytes overlaid on the sliced thymic lobes (indicated as Overlaid) (n = 42 cells for DP and n = 98 cells for SP). See also Table S1. All data are presented as mean ± SD. p values were calculated by Student's two-sample t test. (H) The level of ERK phosphorylation (pERK) analyzed by immunoblotting. The whole thymus and single-cell suspension of the thymus (isolated thymocytes) were prepared for immunoblotting (top). Then, phosphorylation of ERK was analyzed (bottom). The level of ERK is shown as a loading control.

We next attempted to visualize the ERK activity in medullary thymocytes. Because the light scattering in the thymic capsule and cortex prevented us from observing the thymic medulla, sliced thymic lobes were used for the visualization of ERK activity (Figure 3A). In agreement with the previous reports (Korthals et al., 2014, Melichar et al., 2013), we found that thymic dendritic cells (DCs) exhibited bright autofluorescence that could be detected through both BA 520-560 nm and BA 615-675 nm filters (Figure 3B). Therefore, we can distinguish thymic DCs from thymocytes and non-T cells (Figure 3C) and delineate the cortical-medullary junction by the medullary distribution of DCs (Figure 3C, dashed line). In the following analysis, the signals from DCs were excluded by masking. Upon analysis of FRET images, we found that the ERK activity was markedly higher in cortical thymocytes than in medullary thymocytes (Figures 3D and 3E). These and the following FRET/CFP ratio data were pooled from at least two individual mice experiments. The number of cells from each mouse within each group was summarized in Table S1. Because DP and SP thymocytes localized primarily at the cortex and the medulla, respectively, this observation suggested that the level of ERK activity was higher in DP than in SP thymocytes. To confirm this possibility, we isolated thymocytes from the thymus and sorted them into DP and SP thymocytes (Figure 3F, left). Unexpectedly, we failed to detect a significant difference in ERK activity between the DP and SP thymocytes that were isolated by fluorescence-activated cell sorting (FACS) (Figure 3G). To resolve the discrepancy between the observation in the sliced thymic lobes and isolated thymocytes *in vitro*, we next placed DP and SP thymocytes back onto the thymic slice. After at least 3 hr of incubation, during which period the thymocytes migrated down to the thymic tissue, ERK activity in the thymocyte subsets was observed by TPEM (Figure 3F, right). Within the thymus, DP thymocytes showed higher ERK activity than did SP thymocytes (Figure 3G), which recapitulated the observation in the sliced thymic lobes (Figure 3E). We also noticed that the ERK activities in DP and SP thymocytes *in vitro* were significantly lower than those of thymocytes overlaid onto the sliced thymic lobes (Figure 3G). This observation was also confirmed by immunoblotting with anti-phospho-ERK antibody. Phospho-ERK was detected in the whole thymus, but not in the isolated thymocytes (Figure 3H). These results clearly demonstrate that environmental cues in the thymus cause ERK activation in thymocytes and highlight the importance of live-cell imaging under physiological conditions to understand the regulation of ERK signaling in the thymic microenvironment.

### Time-Lapse Imaging Reveals a Negative Correlation between ERK Activity and Cell Motility

**Figure 4:**
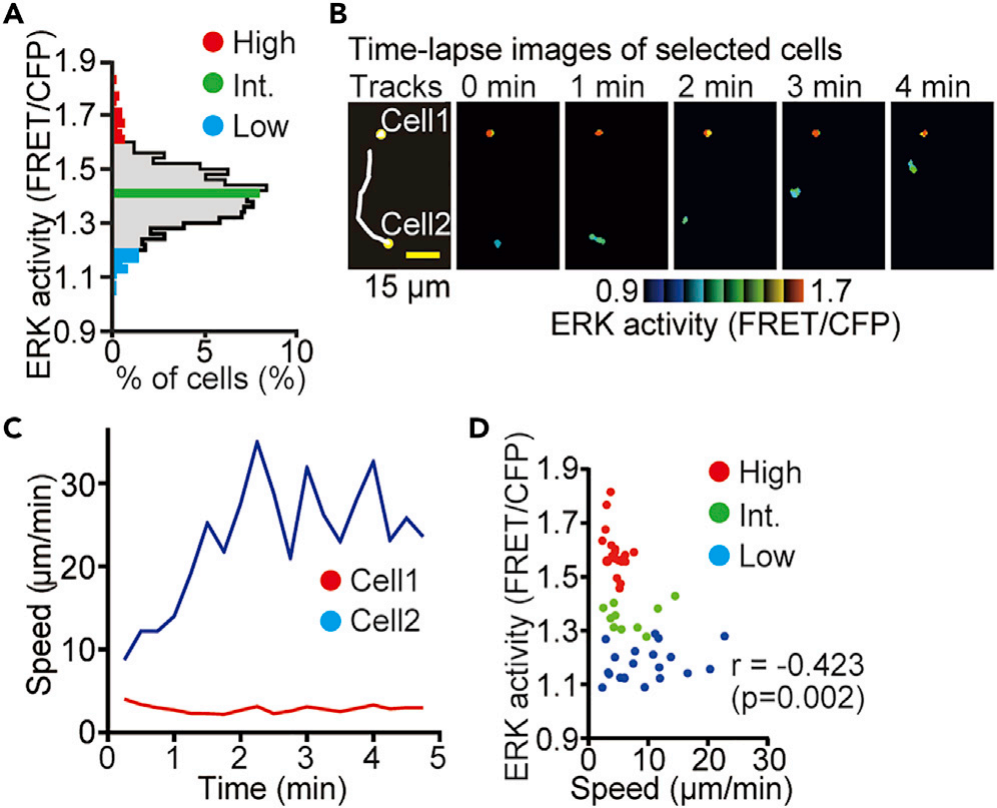
Time-lapse Imaging Reveals a Negative Correlation between ERK Activity and Cell Motility (A) Histogram of the FRET/CFP ratio of thymocytes in the thymic cortex in the sliced thymic lobes. Thymocytes with high ERK activity (top 3% of activity; the 20 cells shown in red), intermediate (int.) ERK activity (10 cells, green), and low ERK activity (bottom 3% of activity, the 20 cells in blue) were categorized into three groups and tracked for the analysis. Data represent the analysis of samples from three individual mice. See also Table S1. (B) Representative time-lapse FRET/CFP ratio images of thymocytes in intensity-modulated display (IMD) mode. (Left) The white lines indicate the cell tracks for the duration of the movie, with the yellow spot indicating the location of the cell at 0 min. (Right) Time-lapse FRET/CFP ratio images in IMD mode. The movie was obtained for 5 min. Scale bar, 15 μm. (C) Time-lapse analysis of the speed of two representative cells shown in (B). To smooth a dataset, the averaged value of five successive time frames was adopted. (D) The relationship between the average FRET/CFP ratio and the average speed (μm/min) of thymocytes. Each dot indicates an individual cell track and is color coded as in panel (B): n = 20 tracks (High); n = 10 tracks (Int.); n = 20 tracks (Low). Data represent the analysis of samples from three individual mice. The relationship between pairs of variables was analyzed using the Pearson correlation analysis.

During the observation of ERK activity in the sliced thymic lobes, we noticed a negative correlation between ERK activity and the motility of thymocytes. To corroborate this observation, we collected 5-min tracks of cortical thymocytes migrating within the sliced thymic lobes. Because the thymus is densely packed with thymocytes and each cell moves stochastically in the 3D space, we were unable to track each cell automatically. For the manual tracking, thymocytes were first categorized into three groups based on the ERK activity at 0 min (Figure 4A). Thymocytes with high ERK activity (top 3% of activity: the 20 cells shown in red), intermediate (int.) ERK activity (the 10 cells shown in green), and low ERK activity (bottom 3% of activity: the 20 cells shown in blue) were selected and tracked (Figures 4B and 4C). We found that thymocytes with high or intermediate ERK activity did not move or moved only a short distance during the observation (Figures 4B–4D). In contrast, thymocytes with low ERK activity moved vigorously within the thymic tissue, suggesting that ERK activation decreased cell motility in the thymic cortex.

### Thymocyte Motility Is Negatively Regulated by ERK Activity

**Figure 5:**
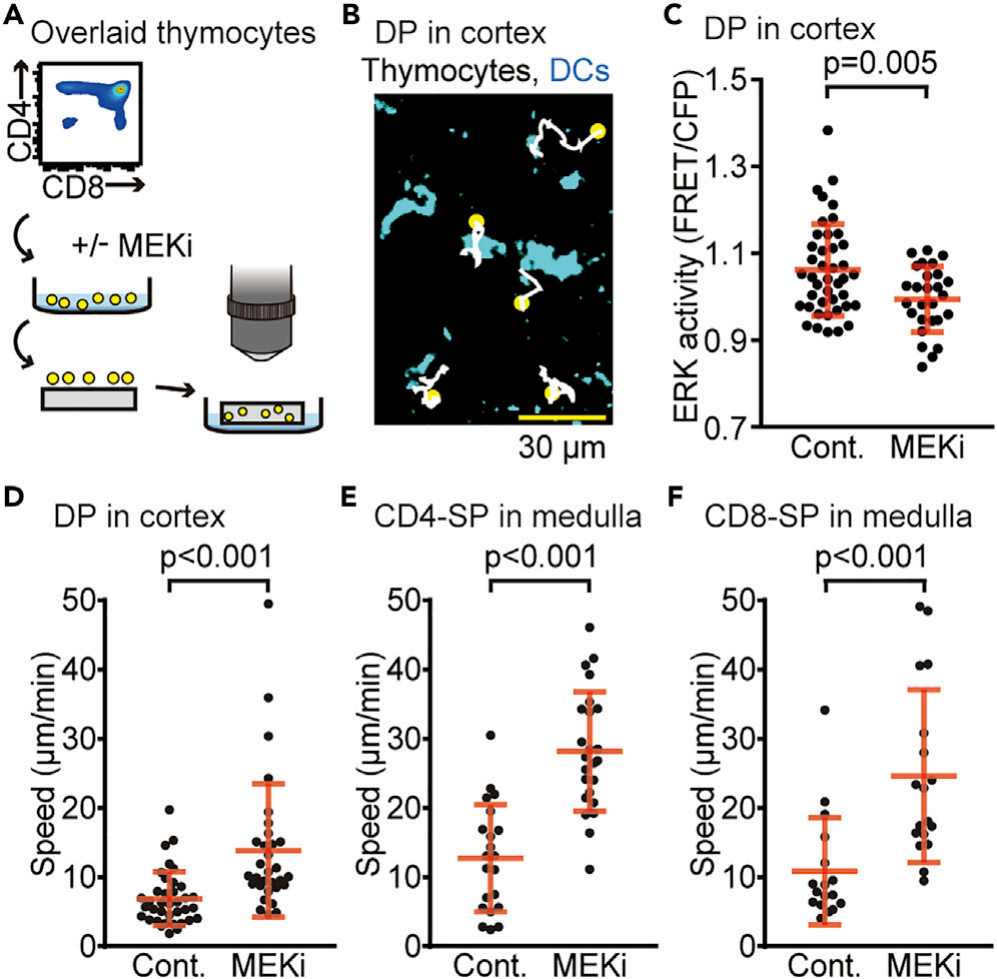
Thymocyte Motility is Negatively Regulated by ERK Activity (A) The experimental design for analyzing the effect of MEK inhibition on thymocyte motility. The sorted subsets of thymocytes from Lck-EKAREV-NLS mice were cultured in the presence or absence of MEKi (10 μM U0126) for 15 min, and overlaid onto sliced thymic lobes obtained from C57BL/6 mice. Thymocytes overlaid onto the sliced thymic lobes were analyzed within 5 hr after MEKi treatment. (B) Representative tracks of DP thymocytes on the sliced thymic lobes. Cell tracks are displayed on the maximum intensity projection of z stack images (128 × 128 μm to a depth of 30 μm). The white lines indicate the cell tracks for the duration of the movie, with the yellow spots indicating where the track started. Cyan regions represent thymic DCs determined by their autofluorescence. The movie was obtained for 30 min. Scale bar, 30 μm. (C) The FRET/CFP ratio of DP thymocytes in the cortex with or without MEKi pretreatment (10 μM U0126 for 15 min). Each dot indicates an individual cell: n = 42 cells (-MEKi); n = 27 cells (+MEKi). Data represent the analysis of samples from three individual mice. See also Table S1. All data are presented as mean ± SD. p value was calculated by Student's two-sample t test. (D–F) The average speed of thymocytes overlaid onto the sliced thymic lobes. DP thymocytes in the cortex (D), CD4-SP in the medulla (E), and CD8-SP in the medulla (F) with or without MEKi pretreatment. Data represent the analysis of samples from three individual mice: n = 37 tracks (DP, -MEKi); n = 31 tracks (DP, +MEKi); n = 21 tracks (CD4-SP, -MEKi); n = 24 tracks (CD4-SP, +MEKi); n = 17 tracks (CD8-SP, -MEKi); n = 18 tracks (CD8-SP, +MEKi). See also Table S1. All data are presented as mean ± SD. p values were calculated by Student's two-sample t test.

To further explore the role of ERK activity in thymocyte motility, sorted subsets of thymocytes were cultured in the presence or absence of an MEK inhibitor, U0126, before transfer onto sliced thymic lobes (Figure 5A). The thymocytes overlaid onto sliced thymic lobes were highly motile and randomly changed direction among thymic DCs (shown in cyan in Figure 5B). As expected, U0126 pretreatment decreased the ERK activity of DP thymocytes in the thymic cortex (Figure 5C) and increased cell motility (Figure 5D). This result supports the idea that high ERK activity suppresses the cell motility of DP thymocytes in the thymic cortex. To examine whether ERK also plays a role in cell motility in the medulla, similar experiments were performed with the CD4-SP and CD8-SP subsets. Again, treatment with U0126 before seeding onto sliced thymic lobes increased cell motility in both the CD4-SP (Figure 5E) and CD8-SP (Figure 5F) subsets. Collectively, these results indicate that thymocyte motility is negatively regulated by ERK activity in both the thymic cortex and the medulla.

### ERK Activity Dynamics Regulates Cell Motility in the CD4-SP Subset

**Figure 6:**
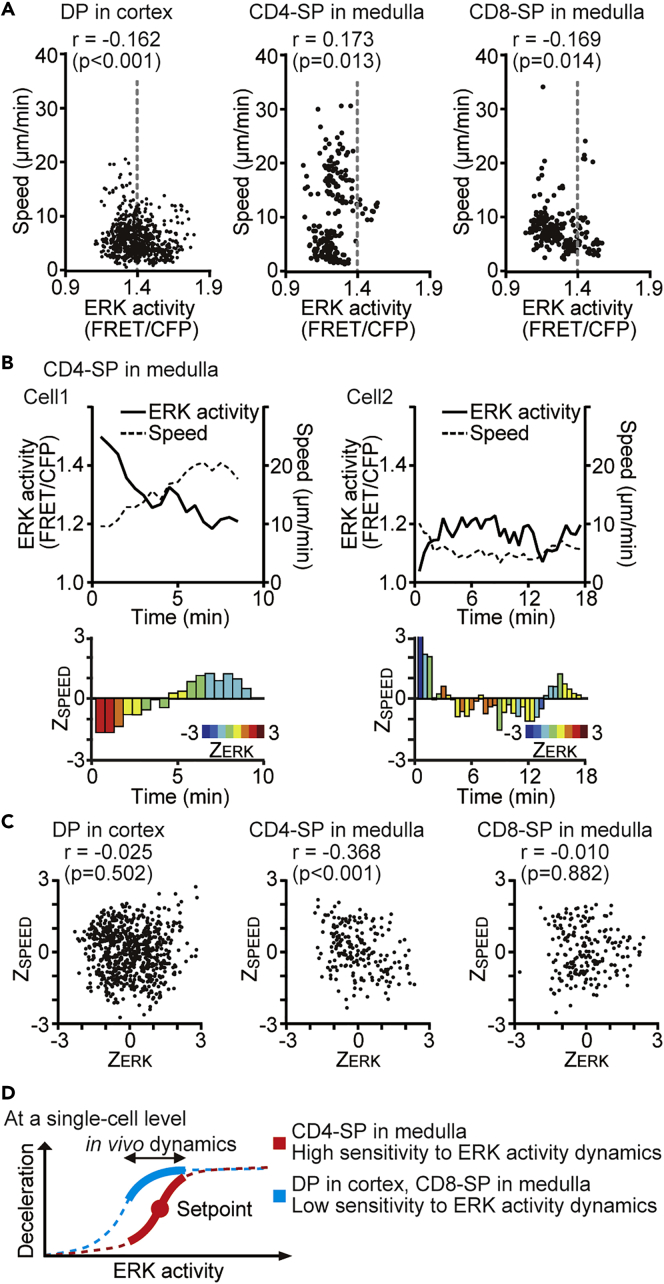
The Deviation of ERK Activity from the Average Regulates Motility in the CD4-SP Subset (A) The relationship between the absolute value of the FRET/CFP ratio and the speed (μm/min) in each subset of thymocytes. The gray dashed line represents the average value of ERK activity in DP thymocytes. Data represent the analysis of samples from three individual mice: n = 37 tracks (DP); n = 21 tracks (CD4-SP); n = 17 tracks (CD8-SP). See also Table S1. The relationship between pairs of variables was analyzed using the Pearson correlation analysis. (B) Representative time-lapse analysis of two CD4-SP cells. ERK activity (black line) and the speed (μm/min) (black dashed line) are shown on the line plot (top) and the deviation of ERK activity (Z_ERK_) and that of speed (Z_SPEED_) are shown on the bar plot (bottom). The color of the bar represents the relative deviation of the ERK activity from the average, and the length of the bar represents the relative deviation of the speed. (C) The relationship between Z_ERK_ and Z_SPEED_ in each subset of thymocytes analyzed in (A). The relationship between pairs of variables was analyzed using the Pearson correlation analysis. (D) Schematic model of the relationship between ERK activity dynamics and thymocyte motility. At the single-cell level, CD4-SP cells respond to the deviation from the set point for ERK activity, whereas other subsets respond to the strength of ERK activity. Thus, CD4-SP cells are more sensitive to the physiological range of ERK activity dynamics than the other subsets.

The migration-promoting effect of the MEK inhibitor indicates that ERK plays a negative role in cell motility. To validate this model without the use of the MEK inhibitor, we performed time-lapse imaging of each subset of thymocytes overlaid onto sliced thymic lobes and examined the correlation between the ERK activity and migration speed (Figure 6A). Albeit weak, a negative correlation was observed between the ERK activity and the motility speed in DP (r = −0.162, p < 0.001) and CD8-SP (r = −0.169, p = 0.014) subsets. Against our expectations, however, the negative correlation was not observed in CD4-SP (r = 0.173, p = 0.013). We speculated that the dynamics of ERK activity, but not the absolute activity at each time point, may regulate the motility of the CD4-SP subset. The data of two cells, one representing high and one representing low ERK activity, are shown in Figure 6B. We can see that the decline of ERK activity correlates with the incline of the cell speed in either case (Figure 6B, top). To corroborate the idea that the ERK activity dynamics regulates the cell motility, we applied *Z* score transformation to the FRET/CFP ratio and motility speed of each cell track (Bazellières et al., 2015, Mrass et al., 2017). With this method, we can analyze the correlation between the deviation of ERK activity (Z_ERK_) and that of speed (Z_SPEED_) in a single cell. We can see a negative correlation between Z_ERK_ and Z_SPEED_ in either cell (Figure 6B, bottom). Analysis of 21 cells obtained from three individual mice revealed that Z_ERK_ was negatively correlated with Z_SPEED_ in the CD4-SP subset (Figure 6C), demonstrating that the ERK activity dynamics indeed had a negative effect on the cell motility of CD4-SP cells. Notably, the correlation between Z_ERK_ and Z_SPEED_ was not observed in DP and CD8-SP subsets (Figure 6C). As the motility speed is larger in CD4-SP than in DP and CD8-SP, this result implies that the role played by ERK may differ between the high- and low-speed ranges. Collectively, these data have shown that ERK activity negatively regulates the motility of DP, CD4-SP, and CD8-SP subsets, although the mode of regulation appears to be different in each cell type. The difference in the mode of regulation implies that, at the single-cell level, CD4-SP cells are more sensitive to the physiological range of ERK activity dynamics than other subsets in the thymic microenvironment (Figure 6D).

### The Intercellular Association between TCR and MHC Class II Molecules Regulates ERK Activity Dynamics in CD4-SP Thymocytes

**Figure 7:**
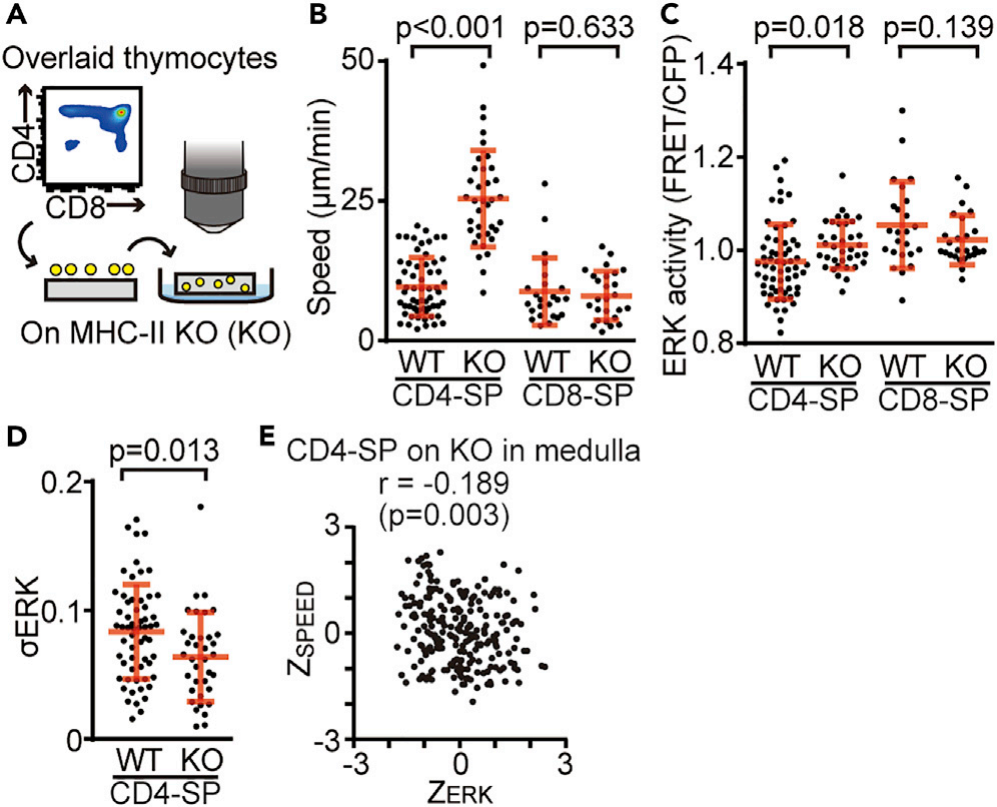
The Intercellular Association between TCR and MHC Class II Molecules Regulates ERK Activity Dynamics in CD4-SP Thymocytes (A) The experimental design for analyzing the effects of TCR-MHC II interactions on ERK activity dynamics and cell motility. The sorted subsets of thymocytes from Lck-EKAREV-NLS mice were overlaid onto sliced thymic lobes obtained from MHC-II KO mice (KO). (B and C) The average speed (B) and the averaged ERK activity (C) of the indicated subsets of thymocytes overlaid onto WT or KO sliced thymic lobes. All data are presented as mean ± SD. p values were calculated by Student's two-sample t test. (D) The standard deviation of ERK activity (σERK) of CD4-SP cells overlaid onto WT or KO sliced thymic lobes. Each dot indicates an individual cell track. Data represent the analysis of samples from two individual mice: n = 60 tracks (CD4-SP on WT slices); n = 36 tracks (CD4-SP on KO slices); n = 24 tracks (CD8-SP on WT slices); n = 26 tracks (CD8-SP on KO slices). See also Table S1. All data are presented as mean ± SD. p value was calculated by Student's two-sample t test. (E) The relationship between Z_ERK_ and Z_SPEED_ in CD4-SP cells overlaid onto KO sliced thymic lobes. The relationship between pairs of variables was analyzed using the Pearson correlation analysis.

The TCR-MHC interaction is known to regulate ERK activity and cell motility (Downward et al., 1990, Dustin et al., 1997), but the direct relationship between the ERK activity regulated by this interaction and cell motility in living tissues remains unknown. To examine the contribution of the intercellular association between TCR and MHC class II molecules to the ERK activity dynamics and motility of CD4-SP in the medulla, we performed time-lapse imaging of CD4-SP and CD8-SP subsets of thymocytes overlaid onto sliced thymic lobes derived from MHC class II knockout (MHC-II KO) mice (Figure 7A). The motility of CD4-SP, but not CD8-SP, was markedly increased when the cells were overlaid onto the thymic slices of MHC-II KO mice (Figure 7B). The ERK activity of CD4-SP was slightly higher than that of the thymocytes overlaid onto WT slices (p = 0.018) (Figure 7C). Meanwhile, the ERK activity of CD8-SP was unchanged by the use of thymic slices of different origins (Figure 7C). Because the ERK activity dynamics regulates cell motility in CD4-SP thymocytes (Figure 6), the fluctuation of ERK activity will reflect the level of motility arrest of CD4-SP. Therefore, we next analyzed the standard deviation of ERK activity in each CD4-SP cell on WT and MHC-II KO thymic slices (Figure 7D). The standard deviation of ERK activity in each single CD4-SP cell on MHC-II KO slices was decreased compared with that of each cell on the WT slices, implying that the disruption of TCR signaling suppress the ERK activity dynamics. Next, to examine whether the mode of motility regulation observed in CD4-SP was affected by the thymic microenvironment of MHC-II KO mice, we examined the relationship between Z_ERK_ and Z_SPEED_ of CD4-SP cells on MHC-II KO slices. Z_ERK_ was negatively correlated with Z_SPEED_ (Figure 7E), indicating that the mode of cell motility regulation observed in CD4-SP was a cell-intrinsic mechanism. Collectively, these results demonstrate that intercellular association between TCR and MHC class II molecules regulates the ERK activity dynamics and cell motility of CD4-SP thymocytes in the medulla.

## Discussion

Here, we described the development of the knockin reporter mice for investigating the relationship between ERK activity and the motility in thymocytes. The reporter mice allowed us to determine that the thymic microenvironment regulates the ERK activity of thymocytes (Figure 3) and thereby modulates the thymocyte motility in a subset-dependent manner (Figures 4 and 5).

Owing to the recombination between CFP and YFP, the development of transgenic mice expressing the FRET biosensors composed of CFP and YFP has been a difficult task (Komatsubara et al., 2015). Transposon-mediated gene insertion into the zygotic genome was found to overcome this problem and was applied to generate a number of transgenic mice expressing the FRET biosensors. However, the expression levels of biosensors in lymphocytes are often insufficient in transgenic mouse lines developed by random transgenesis, including the transposon-mediated method (Figure 1) (Direnberger et al., 2012, Yoshikawa et al., 2016). To overcome the low expression of biosensors in lymphocytes, we chose targeted transgenesis into a permissive locus, such as the *ROSA26* locus, or into the hypoxanthine phosphoribosyltransferase (*HPRT*) locus (Bronson et al., 1996, Zambrowicz et al., 1997), which is neutral for the activity of exogenous enhancers. Among these targets, the *ROSA26* locus offers an open chromatin configuration in all tissues and has been used as a preferred insertion site for ubiquitous expression (Abe and Fujimori, 2013). Indeed, this approach targeting the *ROSA26* locus has been successfully applied to fluorescent reporter protein expression in T cells (Martinez et al., 2015, Tomura et al., 2013). More recently, ubiquitous expression of FRET biosensors for Rho family GTPases was achieved by the insertion of cDNAs into the *Rosa26* locus and the *Hprt* locus (Johnsson et al., 2014, Nobis et al., 2017). Insertion of EKAREV into the ROSA26 locus successfully overcame the low expression of EKAREV in T cells (Figure 1E). Consistent with the previous reports, our results also support that the insertion of a gene of interest into the *Rosa26* locus yielded a strong and ubiquitous expression of the inserted gene without gene silencing (Kong et al., 2014, Zambrowicz et al., 1997).

One advantage of live-cell imaging is that it allows the observation of single-cell dynamics under physiological conditions. Such information of single-cell dynamics has been utilized for understanding intercellular communications, especially in the immune system (Kunz et al., 2018). For example, live-cell imaging has been used to visualize the direct interaction of CD4 and CD8 T cells with DCs after immunization (Bousso and Robey, 2003, Stoll et al., 2002). CD8 T cells were activated after a brief exposure to antigen, whereas CD4 T cells were found to be activated only after multiple and extended encounters with DCs. More recently, biosensors for intracellular Ca^2+^ have been utilized to add mechanistic insights (Okada et al., 2016). The surge in intracellular calcium concentration is an acute marker of the perception of extracellular signals in almost all cell types, including lymphocytes (Clapham, 2007, Feske, 2007, Giorgi et al., 2018), and thus Ca^2+^ sensors are powerful tools for the observation of cell-to-cell communications. On the other hand, to catch the calcium spikes, the intervals of image acquisition should be within a few minutes, which limits prolonged imaging due to phototoxicity. Meanwhile, ERK is an established activation marker of most, if not all, cell surface receptors associated with tyrosine kinases (Arthur and Ley, 2013, Katz et al., 2007, Navarro and Cantrell, 2014). Because ERK activation remains for several minutes to hours after receptor activation, monitoring of ERK activity appears to be more suited to visualize the activation states of immune cells, the period of which ranges from minute to hours. In this study, we established a protocol to visualize the ERK activation states, and thereby the activation of T cells and thymocytes, which will pave the way to understanding the mechanism of immune cell activation in living tissues.

Environmental cues in the thymus cause high basal ERK activity in thymocytes (Figure 2). The environmental cues in the thymic slice could include extracellular matrix (ECM), MHC molecules providing TCR signals, and soluble proteins such as cytokines and chemokines. Among them, ECM would be the most important cue. It is now recognized that ECM is not just a support structure, but a dynamic system that programs cellular functions through physicochemical means (Daley and Yamada, 2013). ECM also serves as a reservoir for chemokines, growth factors, and cytokines (Watt and Huck, 2013). Thus, we speculate that ECMs would be the nature of the environmental cues in the thymus. To verify this idea, we may seed thymocytes and TECs into artificial 3D architecture to recapitulate the environmental cues. TECs can serve as the source of most of the above-mentioned factors, including ECM (Breed et al., 2017, Gameiro et al., 2010). Notably, TECs requires 3D culture. When placed on two-dimensional culture system, TECs start to express markers of terminally differentiated, senescent epithelial cells or even transdifferentiate into skin cells (Bonfanti et al., 2010, Saunders et al., 1995, Sun et al., 1984). Indeed, Poznansky et al. reported that artificial 3D matrix can support TEC survival and thymocyte development (Poznansky et al., 2000). More recently, functional thymus organoids have been successfully constructed by repopulating decellularized 3D thymic scaffolds with isolated TECs in conjunction with bone marrow progenitors (Fan et al., 2015). These developing methods would recapitulate the environmental cues and facilitate our understanding of the unique thymic microenvironment in the future.

Our time-lapse imaging data have revealed similar but distinguishable relationships between the ERK activity and motility speed among DP, CD4-SP, and CD8-SP. First, the MEK inhibitor-induced acceleration of cell motility was clearer for CD4-SP and CD8-SP in the medulla than for DP in the cortex (Figures 5D–5F). This difference may depend on subsets, tissue architecture of the cortex or medulla, or both. In any case, it is evident that ERK activity plays a role in stalling thymocytes in the thymus. Second, ERK activity was negatively correlated with motility speed in CD8-SP, but not in CD4-SP cells (Figure 6A). Third, although significant correlation between ERK activity and motility speed was not detected in CD4-SP, the *Z* score exhibited a negative correlation between the ERK activity and motility, suggesting that the dynamics, but not the absolute value of ERK activity, plays a role in stalling CD4-SP. Based on these findings, we proposed the model that CD4-SP cells are more sensitive to the physiological range of ERK activity dynamics than other subsets at the single-cell level (Figure 6D). This would allow CD4 T cells to stop and interact efficiently with antigen-presenting cells (APCs) when they receive TCR signaling. CD4 T cells require multiple encounters with DCs to be activated, whereas CD8 T cells require only brief exposure (Bousso and Robey, 2003, Stoll et al., 2002). Thus, the high sensitivity to the ERK activity dynamics in regard to the regulation of cell motility would be beneficial for CD4 T cells to search for and interact with multiple APCs efficiently.

ERK has been implicated in the promotion of cell migration in numerous cell types (Huang et al., 2004). However, this is not always the case. Hogstad et al. reported that sustained ERK activation suppressed DC migration (Hogstad et al., 2018). In our experiment, inactivation of ERK activity with MEK inhibitor resulted in the increase of cell motility (Figure 5). Integrins support the adhesion between TECs and thymocytes (Emre et al., 2013, Lannes-Vieira et al., 1993, Ramarli et al., 1998, Savino et al., 2000). Active ERK plays a role in the regulation of integrin-dependent adhesion or cytoskeletal organization (Fincham et al., 2000, Myou et al., 2002, Sawai et al., 2005). Thus, we speculate that inactivation of ERK activity with MEK inhibitor disrupted the integrin-mediated cell adhesion between TECs and thymocytes, which resulted in the increased migration of thymocytes.

The finding that the dynamics of ERK activity regulates cell motility of CD4-SP cells was remindful of homeostasis, a similar regulatory system in which the physiological variables are maintained at a stable level by controlled variables (Cannon, 1929). Although homeostatic systems have been studied primarily with regard to systemically regulated variables, such as the plasma glucose level and plasma osmolality (Bertholdt et al., 2018, Freychet et al., 1988, Oomura et al., 1969), homeostatic systems also operate at the cellular level (Aguilar-Bryan et al., 1995, Potthoff et al., 2011, Raymond et al., 1999). The interplay between motility and the ERK activity dynamics in CD4-SP thymocytes is sure to be another example of a homeostatic system at the cellular level, emphasizing the promising potential of live-cell FRET imaging for studying the role of ERK activity in T cell biology.

Which signal regulates ERK activity dynamics and cell motility of CD4-SP in the medulla? We speculated that TCR-MHC II interactions mediate a transient ERK activation, which exerts a deceleration force on CD4-SP in the thymic medulla. Indeed, disruption of TCR signaling decreased ERK activity dynamics and markedly increased the cell motility of CD4-SP on MHC-II KO thymic slices (Figure 7), suggesting that TCR signaling regulates ERK activity dynamics and cell motility of CD4-SP cells in the medulla. It is quite possible that interactions with the ECM via integrin family molecules also contribute to this ERK activity dynamics in the thymus. Integrins are heterodimeric transmembrane receptors that interact with components of ECM, resulting in the activation of ERK signaling (Chen et al., 1994, Morino et al., 1995, Schlaepfer et al., 1994). Previous studies have shown that impairment in signaling pathways regulating integrin functions resulted in the alteration of normal thymocyte motility (Choi et al., 2014, Ueda et al., 2012, Ueda et al., 2016). Thus, we assume that interactions with ECM via integrin family molecules also regulate ERK activity dynamics and the cell motility of CD4-SP cells in the medulla.

In conclusion, we have developed transgenic mouse lines expressing a FRET biosensor for ERK and unraveled the mode of regulation of cell motility by ERK activity dynamics within the thymic microenvironment. The environmental cues in the thymus activate ERK activity in the thymocytes. The motility in CD4-SP is highly sensitive to a certain physiological range of ERK signaling dynamics compared with that in other subsets. These findings highlight the importance of ERK activity dynamics and their role in controlling cellular functions under physiological conditions, which could not be addressed by conventional biochemical approaches. The importance of understanding the activity dynamics at single-cell resolution will not be limited to ERK. Live imaging with transgenic mice expressing FRET biosensors with high sensitivity for other signaling molecules could be a powerful tool to address these issues and shed light on many physiological and pathological processes.

### Limitation of the Study

The role of the deceleration of thymocytes during development in the thymus is still unclear. Little is known about the molecular mechanisms governing thymocyte motility (Korthals et al., 2014). Korthals et al. reported that thymocytes with a KO mutation in α-PAK-interacting exchange factor showed greatly increased motility, resulting in defective scanning behavior with impaired positive selection in the cortex. Similarly, impairment in Sema3e/plexin D1 signals has also been reported to enhance migration of SP cells in the medulla, resulting in defective negative selection (Choi et al., 2014, Ueda et al., 2016). Collectively, these findings lead us to speculate that the deceleration of thymocytes regulated by ERK activity dynamics supports antigen scanning by confining migration, thereby contributing to the normal T cell selection in the thymus.

## Methods

All methods can be found in the accompanying Transparent Methods supplemental file.

## Supplemental Information

Document S1. Transparent Methods, Figures S1 and S2, and Table S1
