# Supplementary material for: Live-Cell FRET Imaging Reveals a Role of Extracellular Signal-Regulated Kinase Activity Dynamics in Thymocyte Motility
Source: iScience. 2018 Nov 20;10:98–113. doi: 10.1016/j.isci.2018.11.025 (PMC6277225; doi:10.1016/j.isci.2018.11.025)
Supplement: Document S1. Transparent Methods, Figures S1 and S2, and Table S1 [file mmc1.pdf]

ISCI, Volume *10*

Supplemental Information

Live-Cell FRET Imaging Reveals a Role of Extracellular Signal-Regulated Kinase Activity Dynamics in Thymocyte Motility

Yoshinobu Konishi, Kenta Terai, Yasuhide Furuta, Hiroshi Kiyonari, Takaya Abe, Yoshihiro Ueda, Tatsuo Kinashi, Yoko Hamazaki, Akifumi Takaori-Kondo, and Michiyuki Matsuda

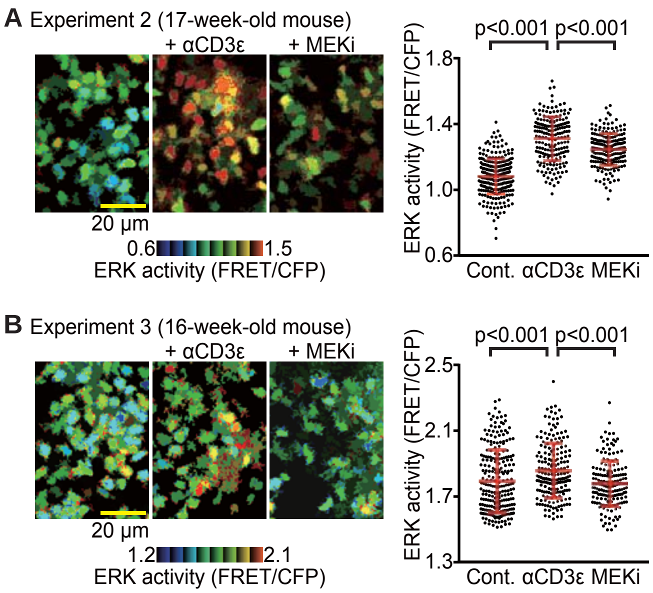

Konishi et al. Fig. S1

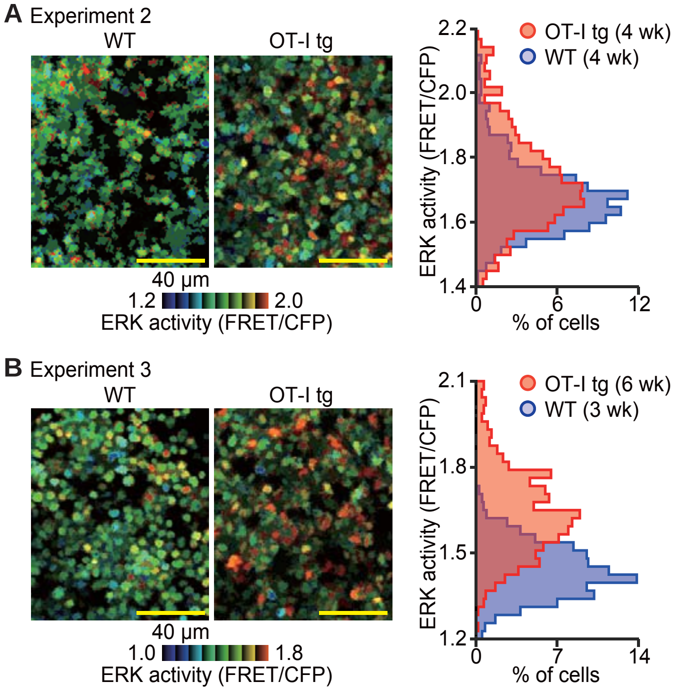

Konishi et al. Fig. S2

Figure S1 (Related to Figure 1). EKAREV-NLS Faithfully Represents ERK Activity in T Cells *In Vivo*.

(A-B) Representative FRET/CFP ratio images shown in IMD mode (left) and the FRET/CFP ratio (right) of the T cells in the paracortex. Scale bar = 20 μm. Dots indicate the FRET/CFP ratio in each T cell. Experiment 2: n = 266 cells (Cont.), n = 191 cells (αCD3ε), and n = 208 cells (MEKi). Experiment 3: n = 248 cells (Cont.), n = 197 cells (αCD3ε), and n = 172 cells (MEKi). The week-old of mice are indicated. p-values were calculated by Student's two-sample *t*-test.

Figure S2 (Related to Figure 2). TCR-MHC Interaction Induces the Upward Shift of ERK Activity.

(A-B) Representative FRET/CFP ratio image of WT thymocytes and OT-I tg thymocytes in the cortex shown in IMD mode (left) and the relative frequency of the FRET/CFP ratio of WT thymocytes (blue) and OT-I tg thymocytes (red) (right). Scale bar = 40 μm. Experiment 2: n = 819 cells (WT), n = 813 cells (OT-I tg). Experiment 3: n = 790 cells (WT), n = 608 cells (OT-I tg).The week-old of mice are indicated.

Table S1. The Number of Cells from Each Mouse within Each Group

Figure	Group	Number of cells on each thymic slice (Each mouse was assigned a letter of the alphabet.)			Total (cells)
		Experiment No.1	Experiment No.2	Experiment No.3	
Figure 3E	Cortex	(A) 162, 186	(C) 220	(D) 108	676
	Medulla	(B) 521	(C) 162	(D) 83	766
Figure 3G	DP (Isolated)	(E) 28	(F) 119	(G) 151	298
	SP (Isolated)	(E) 26	(F) 127	(G) 163	316
	DP (Overlaid)	(H) 11	(I) 26	(J) 5	42
	SP (Overlaid)	(K) 27, 1, 2, 11, 5	(L) 30, 22		98
Figure 4A	Cortex	(A) 162, 186	(C) 220	(D) 108	676
Figure 5C	DP (Cont.)	(H) 11	(I) 26	(J) 5	42
	DP (MEKi)	(M) 16	(N) 10	(O) 1	27
Figure 5D	DP (Cont.)	(P) 6	(Q) 1, 5, 7, 6	(R) 12	37
	DP (MEKi)	(M) 17	(N) 13	(O) 1	31
Figure 5E	CD4 (Cont.)	(P) 1	(S) 13, 5	(T) 2	21
	CD4 (MEKi)	(M) 14	(N) 5	(O) 5	24
Figure 5F	CD8 (Cont.)	(P) 2	(S) 4, 9	(T) 2, 1	17
	CD8 (MEKi)	(M) 8	(N) 8	(O) 2	18
Figure 6	DP	(P) 6	(Q) 1, 5, 7, 6	(R) 12	37
	CD4	(P) 1	(S) 13, 5	(T) 2	21
	CD8	(P) 2	(S) 4, 9	(T) 2, 1	17
Figure 7	CD4 (WT)	(U) 21	(V) 39		60
	CD4 (KO)	(W) 6, 2, 1, 2	(X) 25		36
	CD8 (WT)	(U) 2, 10	(Y) 7, 5		24
	CD8 (KO)	(W) 4	(X) 3, 11, 6, 3		27

Table S1 (Related to Figure 3 to 7). The Number of Cells from Each Mouse within Each Group.

Summarized list showing number of cells from different mice pooled in each group of experiment. Each mouse was assigned a letter of the alphabet.

TRANSPARENT METHODS

Regents and antibodies

U0126 (Sigma-Aldrich, St. Louis, MO) and PD0325901 (Calbiochem, San Diego, CA) were applied as mitogen-activated protein kinase kinase (MEK) inhibitors. Anti-CD3ε antibody (clone 145-2C11) (#100340; Biolegend, San Diego, CA) was used for T-cell activation *in vivo*.

For flow cytometry analysis, anti-mouse CD3 antibody conjugated with PerCP/Cy5.5 (clone 17A2) (#100217; Biolegend), anti-mouse CD4 antibody conjugated with PerCP/Cy5.5 (clone GK1.5) (#100434; Biolegend), anti-mouse CD8a antibody conjugated with APC (clone 53-6.7) (#100712; Biolegend), and anti-mouse CD16/32 antibody (clone 93) (#101320; Biolegend) were used.

For western blotting analysis, anti-phospho-p44/42 MAPK (Erk1/2) (Thr202/Thr204) (D13.14.4E) XP (#4370; Cell Signaling Technology, Danvers, MA) and purified mouse anti-ERK (pan ERK) (16/ERK (pan ERK)) (#610123; BD Biosciences, Franklin Lakes, NJ) antibodies were applied as primary antibodies. Anti-rabbit or anti-mouse secondary IgG antibodies (#926-32220 and #926-32211; LI-COR Biosciences, Lincoln, NE) were used as secondary antibodies.

Plasmids

To generate mouse lines, we constructed pENTR-CAG-lox-tdKeima-EKAREV-NES and pENTR-CAG-lox-tdKeima-EKAREV-NLS plasmids. The Gateway pENTR 2B dual selection vector was purchased from Thermo Fischer Scientific (A10463; Waltham, MA). The CAG promoter and SV40 poly-A sequence were introduced into the pENTR 2B vector by standard subcloning from pT2AL200R175-CAGGS-EGFP (Hitoshi et al., 1991; Urasaki et al., 2006). The woodchuck hepatitis post-transcriptional regulatory element (WPRE) (Klein et al., 2006) was further introduced from CSII-EF-MCS (provided by Hiroyuki Miyoshi, RIKEN BioResource Center Tsukuba, Japan) (Miyoshi et al., 1998). The resulting plasmid was named pENTR-CAG.

The structure of EKAREV was reported previously (Komatsu et al., 2011). Briefly, from the N terminus, EKAREV-NES consists of YPet, which is a FRET-prone variant of YFP, a spacer (Leu-Glu), the WW domain of human Pin1 (a.a. 241-295) used as the ligand domain, a spacer (Gly-Thr), an EV linker, a spacer (Ser-Gly), the substrate peptide (PDVPRTPVDKAKLSFQFP) used as the sensor domain, a spacer (Gly-Gly-Arg), enhanced cyan fluorescent protein (ECFP), and the nuclear export signal (NES) of the HIV-1 rev protein (LPPLERLTLD). In EKAREV-NLS, ECFP and NES were replaced with SECFP, a brighter version of ECFP, and the nuclear localization signal (NLS) of the SV40 large T antigen (PKKKRKV), respectively. Compared to EGFP (GenBank Accession number U76561), the cDNA of Ypet contains mutations of F46L, T65G, V68L, S72A, M153T, V163A, S175G, T203Y, and S208F, the cDNA of ECFP contains Y66W, N146I, M153T, and V163A. The cDNA of SECFP contains additional mutations of K26R, D129G, N164H, and S175G based on the cDNA of ECFP. The DNA encoding tdKeima was cloned from pRSETB tdKeima provided by Atsushi Miyawaki (Riken Brain Science Institute, Wako, Japan) (Kogure et al., 2008) and fused with DNA coding NES, and then with two loxP, one at either end. The resulting DNA was amplified by polymerase chain reaction (PCR) and inserted into the region upstream of the EKAREV-NES or EKAREV-NLS coding DNA. The DNA constructs thus obtained were subcloned into pENTR-CAG vector to generate pENTR-CAG-lox-tdKeima-EKAREV-NES and pENTR-CAG-lox-tdKeima-EKAREV-NLS, respectively.

Generation of *ROSA26* knock-in mice

To develop knock-in reporter mice expressing a genetically-encoded ERK biosensor that was conditionally incorporated into the *ROSA26* locus, *Rosa26* knock-in mice lines, Gt(ROSA)26Sor$^{tm1(CAG\text{-}loxP\text{-}tdKeima\text{-}loxP\text{-}EKAREV\text{-}NES)}$ and Gt(ROSA)26Sor$^{tm1(CAG\text{-}loxP\text{-}tdKeima\text{-}loxP\text{-}EKAREV\text{-}NLS)}$ (hereinafter called R26R-EKAREV-NES and R26R-EKAREV-NLS), were generated by homologous recombination in embryonic stem (ES) cells as described previously (Abe et al.,

2011). In brief, the *ROSA26* targeting vector was constructed using Gateway technology (Thermo Fisher Scientific). The Gateway destination vector, named pMC1-DTA-frt/Neo/frt-ROSA26-DEST, was modified from pMC1-DTA-ROSA26 including the Reading Frame Cassette A from the Gateway Conversion System (Abe et al., 2011). Each cassette in the pENTR-CAG-lox-tdKeima-EKAREV-NES and pENTR-CAG-lox-tdKeima-EKAREV-NLS was recloned into the destination vector using LR clonase of the Gateway technology in order to generate the respective targeting vector. Both targeting vectors were introduced into HK3i ES cells (Kiyonari et al., 2010) by electroporation. G418-resistant colonies were screened for homologous recombinants on each vector by PCR. The recombinant ES cells were injected into eight-cell stage Crl:ICR embryos to generate germline chimera. These offspring mice were crossed twice with B6-Tg (CAG-FLPe) (Kanki et al., 2006) to remove the frt-flanked neo cassette (neomycin-resistance gene with the PGK1 promoter and poly-A signal) (Srinivas et al., 2001). The resulting mouse lines, namely R26R-EKAREV-NES (accession no. CDB0306K: http://www2.clst.riken.jp/arg/reporter_mice.html) and R26R-EKAREV-NLS (accession no. CDB0307K), were then backcrossed with C57BL/6JJmsSlc mice (WT) (Japan SLC, Hamamatsu, Japan) for more than two generations. Offspring were routinely genotyped by PCR with the primers 5’-TCC CTC GTG ATC TGC AAC TCC AGT C-3’ and 5’-AAC CCC AGA TGA CTA CCT ATC CTC C-3’ for the wild type allele, and the primers 5’-GAT CCT CTC GAG CTC GAC TG-3’ and 5’-AAC CCC AGA TGA CTA CCT ATC CTC C-3’ for the R26R allele, yielding 217 bp and 274 bp products, respectively. Further details concerning reporter mouse production will be provided upon request.

Mice

Transgenic mice expressing EKAREV-NES (Eisuke) or EKAREV-NLS (Eisuke-NLS) have been described previously (Kamioka et al., 2012). B6.129S2-H2$^{dIAb1\text{-}Ea}$ (MHC-II KO) mice (Madsen et al., 1999) were provided by Nagahiro Minato (Kyoto University, Kyoto, Japan).

R26R-EKAREV-NES male mice were crossed with B6.FVB-Tg (EIIa-cre) (Lakso et al., 1996) female mice (provided by Mitinori Saitou, Kyoto University, Kyoto, Japan) for the ubiquitous expression of EKAREV-NES. In addition, R26R-EKAREV-NLS mice were crossed with B6.Cg-Tg (Lck-Cre) mice (provided by Nagahiro Minato, Kyoto University, Kyoto, Japan) (Hennet et al., 1995) for T cell specific expression of EKAREV-NLS, resulting in Lck-Cre/R26R-EKAREV-NLS (hereinafter called Lck-EKAREV-NLS) mice. Lck-EKAREV-NLS mice were crossed with C57BL/6-Tg (TcraTcrb) 1100Mjb/J (OT-I) mice (provided by Nagahiro Minato, Kyoto University, Kyoto, Japan) (Hogquist et al., 1994) to obtain OT-I/ Lck-EKAREV-NLS mice. Mice were housed in a specific pathogen-free facility and received a routine chow diet and water *ad libitum*. Male and female mice of 4 to 18 weeks of age were used for the *in vivo* and *in situ* imagings. The animal protocols were reviewed and approved by the Animal Care and Use Committee of Kyoto University Graduate School of Medicine (nos. 16038, 16549, 17033, 17539 and 17539-2) and the Institutional Animal Care and Use Committee (IACUC) of RIKEN Kobe Branch (A2001-03-72).

Flow cytometry analysis

Single-cell suspensions of lymphoid organs were prepared by mechanically dissociating thymic tissue through a 40 µm nylon cell strainer (Corning, Corning, NY) into Hank's balanced salt solution (HBSS) (H6648; Sigma-Aldrich). After centrifugation, cells were resuspended in PBS containing 3% fetal bovine serum (FBS) (SAFC Biosciences, Lenexa, KS) and analyzed and/or sorted with FACS Aria IIu or FACS Aria IIIu (Becton Dickinson, Franklin Lakes, NJ). The following combinations of lasers and emission filters were used for the detection of fluorescence: for the fluorescence of CFP, a 407 nm laser and an ET470/24m filter (Chroma Technology Corp., Bellows Falls, VT), for the sensitized FRET from YFP, a 407 nm laser and a DF530/30 filter (Omega Optical, Brattleboro, VT), for the fluorescence of YFP, a 488 nm laser and an HQ530/30 filter (Omega Optical), for the fluorescence of tdKeima, a 488 nm laser and an

HQ610/20 filter (Omega Optical), for the fluorescence of PerCP/Cy5.5, a 488 nm laser and a DF695/40 filter (Omega Optical), and for the fluorescence of APC, a 633 nm laser and a DF660/20 filter (Omega Optical). Cells were first gated for size and granularity to exclude cell debris and aggregates. For cell sorting, thymocytes were labeled with the antibodies listed in the Regents and antibodies section below, and sorted into complete medium (Iscove's modified Dulbecco's medium (IMDM) (#12440-053; Thermo Fisher Scientific) containing 4% FBS (SAFC Biosciences, St. Louis, MO), 50 µM 2-mercaptoethanol (Nacalai Tesque, Kyoto, Japan), 100 U/ml penicillin, and 100 µg/ml streptomycin (penicillin-streptomycin mixed solution #26253-84; Nacalai Tesque)). Detailed data analysis was performed using FlowJo software (Tree Star, Ashland, OR).

Western blotting

Isolated thymocyte samples were prepared by mechanically dissociating thymic tissue through a 40 µm nylon cell strainer (Corning, Corning, NY) directly into the lysis buffer (see below). Tissue samples of the thymus were immediately harvested into the lysis buffer. The lysis buffer contained 50 mM Tris-HCl (pH 7.5), 150 mM NaCl (Nacalai Tesque), 1% (w/v) Triton X-100 (Nacalai Tesque), 1 mM EDTA (Dojindo, Kumamoto, Japan), 2 mM dithiothreitol (DTT; Wako Pure Chemical Industries, Osaka, Japan), 0.5 mM phenyl methylsulfonyl fluoride (PMSF) (Sigma-Aldrich), 4 µg/mL leupeptin (Sigma-Aldrich), 10 µg/mL aprotinin (Sigma-Aldrich), 50 mM NaF (Kanto Chemical, Tokyo), and 2 mM Na_3VO_4 (Sigma-Aldrich). These samples were kept on ice until being homogenized using a Bioruptor UCD-200TM homogenizer (Cosmo Bio, Tokyo) 8 times for 30 sec. Insoluble material was removed by centrifugation at 20,000 g for 10 min at 4˚C. The supernatant was mixed with 2x sodium dodecyl sulfate (SDS) sample buffer (1M Tris-HCL (pH 6.8), 50% glycerol (Wako Pure Chemical Industries), 10% SDS (Nacalai Tesque), 0.2% bromophenol blue (Nacalai Tesque), and 10% 2-mercaptoethanol (Nacalai Tesque)). After boiling at 95˚C for 5 min, samples were resolved by SDS-PAGE on SuperSep

Ace 12.5% precast gels (Wako Pure Chemical Industries), and transferred to the PVDF membrane (Merck Millipore, Billerica, MA). All antibodies were diluted in Odyssey blocking buffer (LI-COR). Proteins were detected by an Odyssey Infrared Imaging System (LI-COR) and analyzed with the Odyssey imaging software (LI-COR).

Intravital imaging of living organs by two-photon excitation microscopy (TPEM)

Living mice were observed with an FV1200MPE-BX61WI upright microscope (Olympus, Tokyo) equipped with a UPlanSApo 25x/1.05 numerical aperture (NA) water-immersion objective lens (Olympus) and an InSight DeepSee Ultrafast laser (Spectra Physics, Mountain View, CA). The excitation wavelength for CFP was 840 nm. Fluorescent images were acquired with 4 different detector channels using the following filters and mirrors: an infrared (IR)-cut filter, BA685RIF-3 (Olympus), two dichroic mirrors, DM505 and DM570 (Olympus), and four emission filters, FF01-425/30 (Semrock, Rochester, NY) for the second harmonic generation channel (SHG Ch), BA460-500 (Olympus) for the CFP Ch, BA520-560 (Olympus) for the FRET Ch, and 645/60 (Chroma Technology Corp.) for the tdKeima Ch. The inherent autofluorescence of myeloid cells, including dendritic cells (DCs), was also detected with BA460-500, BA520-560, and 645/60 filters, which enabled us to detect thymic DCs. The microscope was equipped with a two-channel GaAsP detector unit and two multialkali detectors. FLUOVIEW software version 4.1a (Olympus) was used to control the microscope and to acquire images, which were saved in the multilayer 16-bit tagged image file format. Acquired images were processed and analyzed with Metamorph software (Universal Imaging, West Chester, PA) as described previously (Kamioka et al., 2012).

Intravital mouse imaging was performed essentially as described previously (Kamioka et al., 2012). For observations of the liver, the small intestine, and the inguinal lymph node, a small vertical incision was made in the abdominal wall to expose the tissues. The exposed tissues were imaged using a fixation system as described previously (Sano et al., 2016). When necessary,

anti-CD3ε antibody (clone 145-2C11) and PD0325901 were injected intravenously during imaging.

Imaging of thymocytes *in situ* by two-photon excitation microscopy

Intact thymic lobes were prepared essentially as described previously (Witt et al., 2005). The thymic lobes of Lck-EKAREV-NLS mice were quickly harvested and separated in the complete medium described above. The lobes were then placed on an imaging specimen holder using tissue adhesive (Leukosan adhesive; BSN Medical Luxembourg, Luxembourg). Then, the lobes were mounted on the bottom of a RC-26 flow chamber (Warner Instruments, Hamden, CT). Pre-warmed RPMI-1640 medium (Thermo Fischer Scientific) bubbled with 95% O_2 and 5% CO_2 was continuously perfused over the slice at a rate of 1 to 2 ml/min. The temperature in the chamber was maintained at 35.5ºC to 37.5ºC using both the chamber and an in-line heater. The specimen was imaged by TPEM as described above.

Sliced thymic lobes were prepared as described previously (Bhakta et al., 2005; Ueda et al., 2012) with some modifications. A thymic lobe obtained from Lck-EKAREV-NLS mice was embedded in low-melting agarose (A0701; Sigma-Aldrich) and sliced every 400 to 800 µm using a vibratome (MA752 Vibroslice, Campden Instruments, Leicestershire, England, or Leica VT1200S, Leica Biosystems, Nussloch, Eisfeld, Germany) and placed onto a Millicell insert (PICM0RG50; Merck Millipore) in a 35 mm plastic petri dish filled with 1 ml of complete medium. The sliced thymic lobe was subsequently mounted on the bottom of a RC-26 flow chamber and imaged in the same manner as intact thymic lobes. Imaging was performed at least 20 µm beneath the cut surface of the slice to avoid the flow effect.

To analyze specific subsets of thymocytes, isolated thymocytes in the suspension were labeled with the antibodies listed in the Regents and antibodies section, and DP, CD4-SP, and CD8-SP subsets were sorted into complete medium. To examine the ERK activity of thymocytes *in vitro*, sorted thymocytes were resuspended in a 35 mm plastic petri dish filled with RPMI-1640

medium and observed by TPEM. Meanwhile, to examine the ERK activity of thymocytes *in situ*, sorted thymocytes were loaded onto sliced thymic lobes obtained from WT or MHC-II KO mice. When needed, 10 µM U0126 (Sigma-Aldrich) was added to the single-cell suspension before being loaded. The cells on the sliced thymic lobes were incubated for 3 to 20 hours at 37ºC/5% CO_2 to allow cells to infiltrate the tissue. After the incubation, the sliced thymic lobe was mounted and imaged as described above.

Thymocytes were observed essentially as described in the Intravital imaging of living organs section. FLUOVIEW software was used to control the microscope and to acquire images, which were saved in the multilayer 16-bit tagged image file format. For thymocytes in the intact thymic lobes, image areas of 254 x 254 µm to a depth of 150 to 200 µm were acquired, with Z steps spaced 1 to 2 µm apart. Time-lapse image areas of 170 x 170 µm at a depth of 60 µm were acquired every 15 seconds for 20 min. For thymocytes in the sliced thymic lobes obtained from Lck-EKAREV-NLS mice, image areas of 100 x 100 or 128 x 128 µm to a depth of 6 µm were acquired every 15 seconds for 5 min, with Z steps spaced 3 µm apart. For thymocytes overlaid on the thymic slices, image areas of 128 x 128 to 254 x 254 µm to a depth of 30 to 40 µm were acquired every 30 seconds for 5 to 30 min, with Z steps spaced 2 or 3 µm apart. Imaging was performed at least 20 µm beneath the cut surface of the slice to avoid the flow effect.

Acquired images were processed with MetaMorph software and analyzed with standard and custom-written MATLAB scripts (MathWorks, Natick, MA) to obtain the 3D coordinates of the cells to analyze migratory behavior as well as the FRET/CFP ratio for ERK activity analysis. Briefly, after removing the area containing signals of inherent autofluorescence, thymocytes were detected as cells with diameters of 2 to 7 µm in the image obtained with the FRET Ch. Then, the x, y coordinates of cell centroids were determined by MetaMorph software. The z coordinate was determined by MATLAB script as the plane showing the highest intensity at the cell centroid point. Thereafter, fluorescent intensities were calculated as the averaged values of fluorescent intensities of nine pixels around the cell centroid at the z plane determined

previously. All movies were created with images processed with median-filter and Auto Align functions (Metamorph software) for noise reduction and drift correction, respectively. From these sets of data, cellular motility parameters and the FRET/CFP ratio were calculated.

Z-score transformation was applied for analysis of the relationship between the deviation of ERK activity and that of migration speed in a single cell. To smooth a data set, the averaged value of five successive time frames was adapted. The formula for calculating the average value of the FRET/CFP ratio (μERK) or the speed (μSPEED), the standard deviation of the FRET/CFP ratio (σERK) or the speed (σSPEED), and the standard score, z, of the FRET/CFP ratio (Z_{ERK}) or the speed (Z_{SPEED}) in each track are given in the following, where ERK(t) or SPEED(t) indicates ERK activity or interval speed at time t, Z_{ERK}(t) or Z_{SPEED}(t) indicates the z score of ERK activity or interval speed at time t, and N indicates the number of time frames of cell tracking.

$$\mu\mathrm{ERK} = \frac{1}{N}\sum_{t=1}^{N} ERK(t)$$

$$\mu\mathrm{SPEED} = \frac{1}{N}\sum_{t=1}^{N} SPEED(t)$$

$$\sigma\mathrm{ERK} = \sqrt{\frac{1}{N}\sum_{t=1}^{N} |ERK(t) - \mu ERK|^2}$$

$$\sigma\mathrm{SPEED} = \sqrt{\frac{1}{N}\sum_{t=1}^{N} |SPEED(t) - \mu\mathrm{SPEED}|^2}$$

$$Z\_(ERK(t)) = ((ERK(t) - \mu\mathrm{ERK}))\ /\ \sigma\mathrm{ERK}$$

$$Z\_(SPEED(t)) = ((SPEED(t) - \mu\mathrm{SPEED}))\ /\ \sigma\mathrm{SPEED}$$

Statistical analysis

Graphing and statistical analysis was performed with MATLAB software (MathWorks) and GraphPad Prism software (GraphPad Software, La Jolla, CA). The statistical differences between the two experimental groups were assessed by Student's two-sample *t*-test. The relationship between pairs of variables was analyzed using the Pearson correlation analysis.

SUPPLEMENTAL REFERENCES

Abe, T., Hiroshi, K., Go, S., Ken‐Ichi, I., Kazuki, N., Shinichi, A., and Toshihiko, F. (2011). Establishment of conditional reporter mouse lines at ROSA26 locus for live cell imaging. genesis 49, 579-590.

Bhakta, N.R., Oh, D.Y., and Lewis, R.S. (2005). Calcium oscillations regulate thymocyte motility during positive selection in the three-dimensional thymic environment. Nature Immunology 6, 143.

Hennet, T., Hagen, F.K., Tabak, L.A., and Marth, J.D. (1995). T-cell-specific deletion of a polypeptide N-acetylgalactosaminyl-transferase gene by site-directed recombination. Proceedings of the National Academy of Sciences 92, 12070-12074.

Hitoshi, N., Ken-ichi, Y., and Jun-ichi, M. (1991). Efficient selection for high-expression transfectants with a novel eukaryotic vector. Gene 108, 193-199.

Hogquist, K.A., Jameson, S.C., Heath, W.R., Howard, J.L., Bevan, M.J., and Carbone, F.R. (1994). T cell receptor antagonist peptides induce positive selection. Cell 76, 17-27.

Kamioka, Y., Sumiyama, K., Mizuno, R., Sakai, Y., Hirata, E., Kiyokawa, E., and Matsuda, M. (2012). Live Imaging of Protein Kinase Activities in Transgenic Mice Expressing FRET Biosensors. Cell Structure and Function 37, 65-73.

Kanki, H., Suzuki, H., and Itohara, S. (2006). High-efficiency CAG-FLPe Deleter Mice in C57BL/6J Background. Experimental Animals 55, 137-141.

Kiyonari, H., Kaneko, M., Abe, S., and Aizawa, S. (2010). Three inhibitors of FGF receptor, ERK, and GSK3 establishes germline-competent embryonic stem cells of C57BL/6N mouse strain with high efficiency and stability. genesis 48, 317-327.

Klein, R., Ruttkowski, B., Knapp, E., Salmons, B., Günzburg, W.H., and Hohenadl, C. (2006). WPRE-mediated enhancement of gene expression is promoter and cell line specific. Gene 372, 153-161.

Kogure, T., Kawano, H., Abe, Y., and Miyawaki, A. (2008). Fluorescence imaging using a fluorescent protein with a large Stokes shift. Methods 45, 223-226.

Komatsu, N., Aoki, K., Yamada, M., Yukinaga, H., Fujita, Y., Kamioka, Y., Matsuda, M., and Weis, K. (2011). Development of an optimized backbone of FRET biosensors for kinases and GTPases. Molecular Biology of the Cell 22, 4647-4656.

Lakso, M., Pichel, J.G., Gorman, J.R., Sauer, B., Okamoto, Y., Lee, E., Alt, F.W., and Westphal, H. (1996). Efficient in vivo manipulation of mouse genomic sequences at the zygote stage. Proceedings of the National Academy of Sciences 93, 5860-5865.

Madsen, L., Labrecque, N., Engberg, J., Dierich, A., Svejgaard, A., Benoist, C., Mathis, D., and Fugger, L. (1999). Mice lacking all conventional MHC class II genes. Proceedings of the National Academy of Sciences 96, 10338-10343.

Miyoshi, H., Blömer, U., Takahashi, M., Gage, F.H., and Verma, I.M. (1998). Development of a Self-Inactivating Lentivirus Vector. Journal of Virology 72, 8150-8157.

Sano, T., Takashi, K., Hiromitsu, N., Atsushi, S., Takuya, H., Yuji, K., S., L.L., Osamu, O., and Michiyuki, M. (2016). Intravital imaging of mouse urothelium reveals activation of extracellular signal ‐ regulated kinase by stretch ‐ induced intravesical release of ATP. Physiological Reports 4, e13033.

Srinivas, S., Watanabe, T., Lin, C.-S., William, C.M., Tanabe, Y., Jessell, T.M., and Costantini, F. (2001). Cre reporter strains produced by targeted insertion of EYFP and ECFP into the ROSA26 locus. BMC Developmental Biology 1, 4.

Ueda, Y., Katagiri, K., Tomiyama, T., Yasuda, K., Habiro, K., Katakai, T., Ikehara, S., Matsumoto, M., and Kinashi, T. (2012). Mst1 regulates integrin-dependent thymocyte trafficking and antigen recognition in the thymus. Nature Communications 3, 1098.

Urasaki, A., Morvan, G., and Kawakami, K. (2006). Functional Dissection of the Tol2 Transposable Element Identified the Minimal cis-Sequence and a Highly Repetitive Sequence in the Subterminal Region Essential for Transposition. Genetics 174, 639-649.

Witt, C.M., Raychaudhuri, S., Schaefer, B., Chakraborty, A.K., and Robey, E.A. (2005). Directed Migration of Positively Selected Thymocytes Visualized in Real Time. PLOS Biology 3, e160.
